# OR7-002 – Pyrin 577 mutations in dominant autoinflammation

**DOI:** 10.1186/1546-0096-11-S1-A103

**Published:** 2013-11-08

**Authors:** M Stoffels, A Szperl, A Simon, MG Netea, TS Plantinga, M van Deuren, S Kamphuis, H Lachmann, E Cuppen, WP Kloosterman, J Frenkel, CC van Diemen, C Wijmenga, M van Gijn, JW van der Meer

**Affiliations:** 1 Department of General Internal Medicine, Radboud University Nijmegen Medical Centre, Nijmegen, the Netherlands; 2 Nijmegen Centre for Infection, Inflammation and Immunity (N4i), Nijmegen, the Netherlands; 3 Nijmegen Centre for Molecular Life Sciences, Nijmegen, the Netherlands; 4 Department of Genetics, UMC Groningen, Groningen, the Netherlands; 5 Sophia Children’s Hospital, Erasmus UMC, Rotterdam, the Netherlands; 6 Division of Medicine, UK National Amyloidosis Centre, UCLMS, London, UK; 7 Department of Medical Genetics, UMC Utrecht, Utrecht, the Netherlands; 8 Division of Pediatrics, UMC Utrecht, Utrecht, the Netherlands

## Introduction

Autoinflammatory disorders are disorders of the innate immune system. Standard genetic testing provided no correct diagnosis in a female patient from a non-consanguineous family of British descent with a colchicine-responsive autosomal dominant periodic fever syndrome.

## Objectives

We aimed to unravel the genetic cause of the symptoms in this family.

## Methods

Whole exome sequencing was used to screen for novel sequence variants, which were validated by direct Sanger sequencing. Ex-vivo stimulations with peripheral blood mononuclear cells were done to study the functional consequences of the mutation. mRNA and cytokine levels were measured by q-PCR and ELISA, respectively.

## Results

Whole exome sequencing revealed a novel missense sequence variant, not seen in around 6800 controls, mapping to exon 8 of the *MEFV* gene (c.1730C>A; p.T577N), co-segregating perfectly with disease in this family. Other mutations at the same amino acid (c.1730C>G; p.T577S; c.1729A>T; p.T577S) were found in a family of Turkish descent, with autosomal dominant inheritance of FMF-like phenotype, and a Dutch patient, respectively. Moreover, a mutation (c.1729A>G; p.T577A) was detected in 2 Dutch siblings, suffering from episodes of inflammation of varying severity not resembling FMF. PBMCs from one patient of the index family revealed increased basal IL-1β mRNA levels and cytokine responses after LPS stimulation. Responses normalized under colchicine treatment.

## Conclusion

Heterozygous mutations at amino acid position 577 of pyrin can induce an autosomal dominant autoinflammatory syndrome. This suggests that T577, located in front of the C-terminal B30.2/SPRY domain, is crucial for pyrin function.

## Disclosure of interest

M. Stoffels: None declared, A. Szperl: None declared, A. Simon Consultant for: Novartis and Swedish Orphan Biovitrum, M. Netea: None declared, T. Plantinga: None declared, M. van Deuren: None declared, S. Kamphuis: None declared, H. Lachmann: None declared, E. Cuppen: None declared, W. Kloosterman: None declared, J. Frenkel Consultant for: Novartis and Swedish Orphan Biovitrum, C. van Diemen: None declared, C. Wijmenga: None declared, M. van Gijn: None declared, J. van der Meer Consultant for: Novartis and Swedish Orphan Biovitrum

